# Challenges with Approved Targeted Therapies against Recurrent Mutations in CLL: A Place for New Actionable Targets

**DOI:** 10.3390/cancers13133150

**Published:** 2021-06-24

**Authors:** Irene López-Oreja, Heribert Playa-Albinyana, Fabián Arenas, Mónica López-Guerra, Dolors Colomer

**Affiliations:** 1 Experimental Therapies in Lymphoid Neoplasms, Institut d’Investigacions Biomèdiques August Pi i Sunyer (IDIBAPS), 08036 Barcelona, Spain; ilopezor@clinic.cat (I.L.-O.); hplaya@clinic.cat (H.P.-A.); farenas@clinic.cat (F.A.); lopez5@clinic.cat (M.L.-G.); 2 Centro de Investigación Biomédica en Red en Oncología (CIBERONC), 28029 Madrid, Spain; 3 Centre for Genomic Regulation (CRG), The Barcelona Institute of Science and Technology, 08003 Barcelona, Spain; 4 Universitat Pompeu Fabra, 08005 Barcelona, Spain; 5 Hematopathology Section, Hospital Clínic, University of Barcelona, 08036 Barcelona, Spain

**Keywords:** chronic lymphocytic leukemia, toll-like receptor (TLR), MAPK, NOTCH1, SF3B1

## Abstract

**Simple Summary:**

Chronic lymphocytic leukemia harbors a high degree of genetic variability and interpatient heterogeneity. Some of the genetic alterations have an impact on the disease’s prognosis and evolution, but few data exist about the response to new approved targeted therapies in patients carrying recurrent mutations other than *TP53*. In this review, we present the knowledge about the impact of these new genetic alterations in the treatment response together with the possibility to use new actionable targets.

**Abstract:**

Chronic lymphocytic leukemia (CLL) is characterized by a high degree of genetic variability and interpatient heterogeneity. In the last decade, novel alterations have been described. Some of them impact on the prognosis and evolution of patients. The approval of BTK inhibitors, PI3K inhibitors and Bcl-2 inhibitors has drastically changed the treatment of patients with CLL. The effect of these new targeted therapies has been widely analyzed in *TP53*-mutated cases, but few data exist about the response of patients carrying other recurrent mutations. In this review, we describe the biological pathways recurrently altered in CLL that might have an impact on the response to these new therapies together with the possibility to use new actionable targets to optimize treatment responses.

## 1. Introduction

In the last decade, genomic and epigenomic studies have unravel novel alterations that play an important role in the prognosis and evolution of chronic lymphocytic leukemia (CLL) [1,2,3,4,5,6], revealing CLL’s genetic and interpatient heterogeneity. The behavior of the disease is influenced by microenvironmental signals that regulate the proliferation and survival of CLL cells [7]. Two major molecular CLL subgroups have been identified according to the mutational status of the immunoglobulin (Ig) heavy-chain variable region (IGHV) genes. Those harboring unmutated IGHV genes (U-CLL, ≥98% identity with the germline) originate from B cells that have not experienced the germinal center and those with mutated IGHV genes (M-CLL, <98% identity with the germline) originate from post-germinal center B cells [8,9,10]. Furthermore, approximately one third of CLL cases present virtually identical Ig rearrangements, known as stereotypes [11]. Some of these subsets have prognostic value [12] and recently a single point mutation in IGLV3-21 (R110-mutated IGLV3-21) has been associated with an aggressive biological subtype of CLL [13,14].

The mutational landscape of CLL is very heterogeneous, being *NOTCH1*, *SF3B1*, *TP53* and *ATM* the genes that are mutated in more than 5% of cases [2,5]. Recurrent mutations can be grouped in 8 main cellular pathways: DNA damage response (*ATM*, *TP53*, *POT1*); Notch signaling (*NOTCH1*, *FBXW7*); RNA splicing and metabolism (*SF3B1*, *U1*, *XPO1*, *DDX3X*, *RPS15*); B-cell receptor (BCR) and Toll-like receptor (TLR) signaling (*MYD88*, *PAX5*, *KLHL6*, *BCOR, TLR2*, *IKZF3*); MAPK-ERK pathway *(BRAF, KRAS, NRAS, EGR2*); NF-κB signaling (*BIRC3*, *NFKB2*, *NFKBIE*, *TRAF2*, *TRAF3*); chromatin modification (*CHD2*, *SETD2*, *KMT2D*, *ASXL1*) and cell cycle (*ATM*, *TP53*, *CCND2, CDKN1B*, *CDKN2A*) [7]. Furthermore, the number of driver alterations affects the clinical behavior, being the worst prognosis in patients with higher number of driver aberrations [2,15]. The heterogeneity between patients may be influenced by: (a) the cell of origin: U-CLL present more driver mutations than M-CLL and some mutations appear mainly in one of the two major molecular subgroups (e.g., *MYD88* and *PAX5* in M-CLL and *U1*, *NOTCH1*, *POT1* in U-CLL) [2,5]; (b) the age of CLL patients (e.g., young patients carry more frequently *MYD88* mutations) [1,16], (c) the presence of subclonal mutations that may be acquired during disease evolution [5,15,17] and (d) the course of the disease: some mutations impact in the need of treatment (*SF3B1*, *POT1*, *ATM*) and others (*TP53*, *BIRC3*, *MAP2K1, NOTCH1*) are more frequent at relapse after chemoimmunotherapy [7,18,19]. At epigenetic level, few patients carry mutations in chromatin remodeler genes [2,5]. CLL cells are hypomethylated and most of the differences observed between U-CLL and M-CLL are related to different cell of origin. The most important changes are restricted to few transcription factor binding sites and enhancers controling important genes implicated in CLL pathogenesis such as BCR and NF-κB signaling [20].

The treatment of patients with CLL has evolved in the last years from conventional chemotherapeutic agents (e.g., fludarabine, fludarabine plus cyclophosphamide (FC), chlorambucil or bendamustine) combined with anti-CD20 antibodies (rituximab or obinutuzumab), to novel targeted agents. The targeted drugs currently approved by both the US Food and Drug Administration (FDA) and the European Medicines Agency (EMA) are the Bruton’s tyrosine kinase (BTK) inhibitors ibrutinib and acalabrutinib, the Bcl-2 inhibitor venetoclax [21] and the phosphatidylinositol 3-kinase (PI3K) inhibitors idelalisib and duvelisib [22]. These drugs have shown higher clinical responses compared to standard chemoimmunotherapy and all of them seem to act in a TP53 independent manner. Due to the prognostic and therapeutic impact of *TP53* alterations (17p deletion and *TP53* mutation) in CLL, the response of *TP53*-mutated cases to the new agents has been widely analyzed (summarized in Table 1) and recently reviewed [23]. On the contrary, few data exist about the effect of other recurrent mutations in the response to the new approved targeted therapies (Table 1). In this way, the effect of other mutated genes relevant for the DNA damage response pathway such as *ATM*, located at 11q region, a region frequently deleted in CLL cases, has not been widely explored, although it is accepted that 11q alterations are associated with unfavorable prognosis [24]. Another gene located at the minimal deleted 11q region is *BIRC3*. *BIRC3* encodes c-IAP2, a member of the human Inhibitors of Apoptosis Proteins (IAPs) family [25] that acts as a negative regulator of non-canonical NF-κB signaling [26]. *BIRC3* deletions coexist with *ATM* deletions, both have prognostic value in CLL [27,28,29], being patients with a biallelic alteration (deletion and mutation) those with a significantly shorter time to first treatment (TTFT) [30]. *BIRC3* alterations are associated with fludarabine-chemoresistance and adverse prognosis [29], but few information exist about the response to new therapies (Table 1). Mutations in key genes of the alternative NF-kB pathway, such as *BIRC3*, confer resistance to BTK inhibitors in mantle cell lymphoma (MCL) [31,32], that can be overcome with the addition of NIK inhibitors [33]. Furthermore, IAP antagonists, such as SMAC mimetics, have showed activity in CLL cells but not specifically in cases with BIRC3 alterations [34].

In addition, many other mutations have shown a prognostic value, which impacts on the patient’s outcome, regardless of the treatment received. But only *TP53* and *NOTCH1* have a predictive impact, as it has been demonstrated in comparative trials that some treatments have different effect in the presence of these mutations [18,35,36] (Table 1). All this knowledge together with the characterization of the molecular effects of recurrent mutations, foster the development of direct target inhibitors leading to a precision medicine [19,37]. However, the design of direct and specific inhibitors is challenging. In particular, it is very difficult to directly target TP53 due to its structure and nuclear localization [38]. In this context, molecules able to restore the physiological function of TP53, or strategies to potentiate TP53 function by using compounds that bind to MDM2 in the p53-binding pocket have been developed [23]. In CLL, patients with subclonal TP53 mutations might benefit from treatment with these compounds [39]. In addition, other proteins are able to regulate TP53 expression, for example, the XPO1 inhibitor selinexor, which enhances p53 nuclear retention and induces the transcription of TP53 target genes in CLL cells [40]. Other strategies include the use of inhibitors of the checkpoint kinase 1 (CHK1), taking into account that *TP53*-mutated cells lack the G1/S checkpoints and are more vulnerable to this inhibition [41] or the use of compounds inducing reactive oxygen species (ROS) irrespective of p53 status [42]. In this review, we will focus on altered genes/pathways that play a role in the response of new approved therapies in CLL, such as the TLR, MAPK and Notch signaling pathways as well as RNA splicing that can be tackled with new agents to improve the efficacy of current therapy for CLL.

**Table 1:** Effect of the main recurrent mutations on the response to different treatment strategies in CLL.

Mutation		Drug/s	Trial	Response to the Treatment in the Mutated Group	Ref.
*TP53*		FCR vs. FC	CLL8	Worse PFS and OS with both treatments	[43]
		Lenalidomide		Worse OR and PFS in TN and R/R	[44]
		Ibrutinib		Shorter PFS, OS	[45,46]
		Ibrutinib vs. ofatumumab	RESONATE Phase III	Shorter PFS with ibrutinib	[47]
		Ofatumumab vs. OfIde		Similar PFS with OfIde	[48]
		IdeR followed by idelalisib		Similar PFS	[49]
		Duvelisib	DUO extension	Similar PFS	[50]
		Venetoclax		Shorter duration of response	[51]
		VenOb		Shorter PFS	[52]
		(Bendamustine) + VenOb	CLL2-BAG	Lower MRD negativity rates	[53]
		VenR vs. BR	MURANO Phase III	Higher MRD positivity rates at EOT	[54]
		Chl vs. OfChl	COMPLEMENT 1	Worse PFS and OS with both treatments	[24]
		ObChl vs. IbrOb	iLLUMINATE	Similar PFS to overall population with IbrOb	[55]
		ObChl vs. VenOb	CLL14	Lower ORR with ObChl, independent prognostic factor for PFS with both treatments	[56]
		ObChl vs. acalabrutinib vs. ObAca	ELEVATE-TN	PFS benefit with ObAca	[57]
		Acalabrutinib	ACE-CL-001	Similar OR rate regardless del(17p) status	[58]
		Acalabrutinib vs. IdeR vs. BR	ASCEND	PFS benefit with acalabrutinib	[59]
*MYD88*		Chl vs. OfChl	COMPLEMENT 1	No effect in PFS	[24]
MAPKs		Fludarabine vs. FC vs. Chl	CLL4	Independent markers of poor OS	[60]
		PI3K inhibitors		Mutations enriched in non-responder subgroup	[61]
	*BRAF*	VenR vs. BR	MURANO Phase III	Higher MRD positivity rates at EOCT and at EOT	[54]
	*KRAS*	Lenalidomide		Worse OR in TN and R/R	[44]
		Chl vs. ChlR vs. ObChl	CLL11	Non response to chemoimmunotherapy	[62]
*NOTCH1*		FCR vs. FC	CLL8	No benefit from the addition of rituximab to FC	[43]
		Chl vs. OfChl	COMPLEMENT 1	Reduced ofatumumab efficacy	[24]
		Ibrutinib vs. ofatumumab	RESONATE Phase III	Reduced ofatumumab efficacy	[63]
		Ibrutinib		Shorter PFS and OS	[64]
		Venetoclax		Shorter duration of response	[51]
		VenR vs. BR	MURANO Phase III	Higher MRD positivity rates at EOT	[54]
		ObChl vs. VenOb	CLL14	Shorter PFS with ObChl, not statistically significant with VenOb	[56]
*SF3B1*		FCR vs. FC	CLL8	Worse PFS with both treatments	[43]
		Lenalidomide		Worse OS and PFS in R/R	[44]
		Chl vs. OfChl	COMPLEMENT 1	Worse PFS in both arms (also in low VAF patients)	[24]
		Ibrutinib vs. ofatumumab	RESONATE Phase III	Trend to shorter PFS not statistically significant	[47]
		Ibrutinib		Mutation enriched in postreatment samples	[65]
		Ibrutinib vs. acalabrutinib		In patients with RT, *SF3B1* mutations were more frequent than BTK mutations	[66]
		Venetoclax		Shorter duration of response	[51]
		ObChl vs. VenOb	CLL14	Independent prognostic factor for PFS with ObChl	[56]
*BIRC3*		Fludarabine vs. FC vs. Chl	CLL4	Shorter PFS and OS	[60]
		FCR		Shorter PFS	[29]
		Chl vs. OfChl	COMPLEMENT 1	Similar PFS and OS	[24]
		Ibrutinib vs. ofatumumab	RESONATE Phase III	Similar PFS	[63]
		Ibrutinib		Mutation enriched in postreatment samples	[65]
		VenR vs. BR	MURANO Phase III	Higher MRD positivity rates at EOCT, shorter PFS with BR	[54,67]
		ObChl vs. VenOb	CLL14	Independent prognostic factor for PFS with ObChl	[56]

BR: bendamustine + rituximab, Chl: chlorambucil, ChlR: chlorambucil + rituximab, DOR: duration of response, EOCT: end of combination therapy, EOT: end of treatment, FC: fludarabine + cyclophosphamide, FCR: fludarabine + cyclophosphamide + rituximab, GClb: obinutuzumab + chlorambucil, IbrOb: ibrutinib + obinutuzumab, IdeR: idelalisib + rituximab, MRD: minimal residual disease, ObAca: obinutuzumab + acalabrutinib, ObChl: obinutuzumab + chlorambucil, OfChl: ofatumumab + chlorambucil, OfIde: ofatumumab + idelalisib, OS: overall survival, OR: overall response, PFS: progression free survival, R/R: relapsed/refractory patients, RT: Richter transformation, TN: treatment naïve, VAF: variant allele frequency, VenOb: venetoclax + obinutuzumab, VenR: venetoclax + rituximab.

## 2. TLR Signaling

Toll-like receptors (TLRs) are part of the innate immune system capable to identify pathogen-associated molecular patterns (PAMPs) and damage-associated molecular patterns (DAMPs) [68]. PAMPs and DAMPs triggers TLRs transduction and initiate innate and adaptive immune responses to eliminate pathogens and repair the damaged tissues. TLR signaling is necessary together with BCR recognition and T-cell interaction for B-cell activation [69,70].

A total of ten TLRs are expressed in human immune and non-immune cells. They are located in the cell surface (e.g., TLR1, TLR2 and TLR4-6) or in endolysosomes (TLR3 and TLR7-9) [71]. When TLRs recognize some PAMPs or DAMPs, a cell signaling cascade is initiated throughout the recruitment of an adaptor protein. There are two types of adaptor proteins: myeloid differentiation factor 88 (MyD88) and TIR-domain containing adaptor molecule (TRIF). MyD88 forms the Myddosome together with interleukin 1 receptor associated kinase 4 (IRAK4) and kinase 1 and 2 (IRAK1/2) [72]. The Myddosome produces pro-inflammatory cytokines (e.g., IL-1, IL-6, IL-12 and TNF) and via TNF receptor-associated factor 6 (TRAF6), activates different molecular pathways.

**Figure 1:**
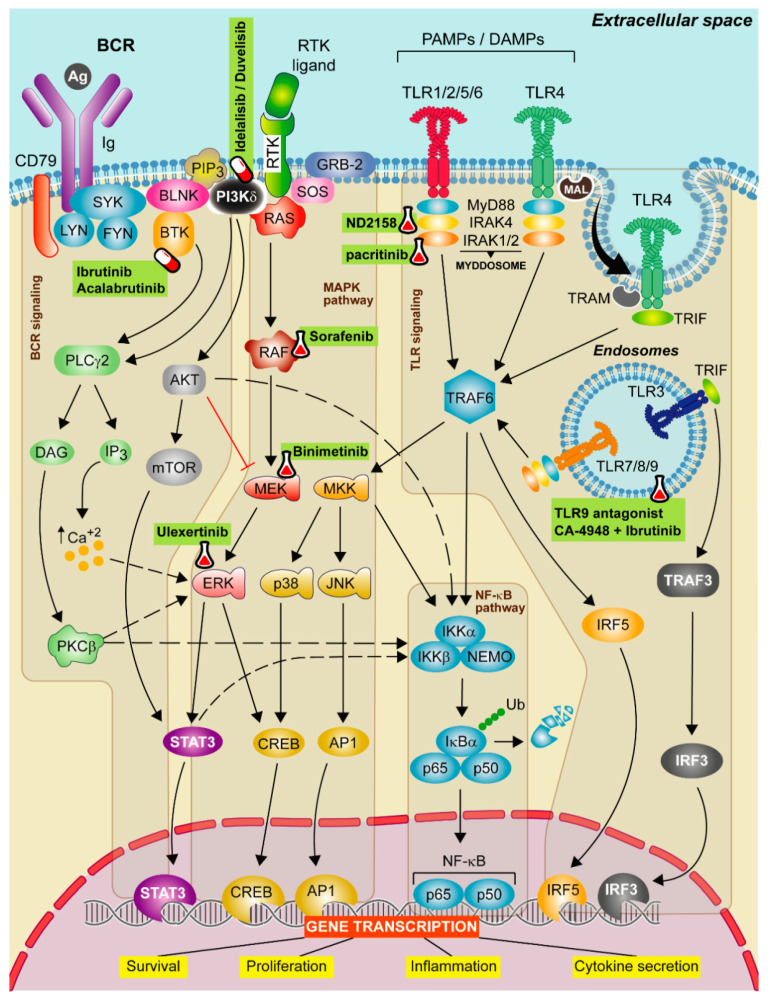
Therapeutic strategies targeting TLR, BCR, and MAPK signaling in CLL. Schematic representation of the main activation events in the B-cell receptor (BCR), mitogen-activated protein kinase (MAPK) and Toll-like receptor (TLR) signaling pathways. BCR signaling activation iniciates when the antigen binds to the receptor leading to CD79 phosphorylation through LYN and SYK tyrosine kinases forming a signalosome that includes the BLNK, the BTK and the PI3Kδ. These proteins transduce signals to calcium signaling modulator PLCγ2. PLCγ2 degrades the PIP_2_ into IP_3_ and DAG, releasing calcium from endoplasmic reticulum (ER) and promoting the activation of the PKCβ. Then, PKCβ activates the NF-κB pathway and ERK signaling. The canonical MAPK pathway is activated after a ligand binds to the RTK. Then, RAS protein recruits adaptor proteins, such as GRB-2 and SOS and promotes the formation of RAF dimers. This induces the MEK-ERK cascade concluding with the translocation of STAT3 and CREB transcription factors to the nucleus. In contrast, the non-canonical MAPK pathway is activated by TRAF6, then MKK is phosphorylated and this leads to the activation of NF-κB pathway and to p38 and JNK phosphorylation that promote the translocation of CREB and AP1 transcriptions factors to the nucleus. All these events favor cell survival, cell proliferation and cytokine secretion signals. Finally, TLR signaling pathway is activated by TLRs located on either the cell membrane or endolysosomal membranes. These receptors are capable to detect PAMPs which initiate the downstream signaling, mediated by two main adaptor proteins: MyD88 and/or TIR domain containing adapter molecule (TRIF) dependent pathways. MyD88 is recruited to the TIR domain of TLRs inducing IRAK1/2 and IRAK4, jointly with MyD88, to form the Myddosome. Aside from MyD88-dependent signal transduction, TLR3 and TLR4 use the TRIF adaptor protein. TLR3 binds directly to TRIF whereas TLR4 needs TRAM. TLR3 and 4 bind to TRAF3 which induce the expression of IRF3, promoting the synthesis of type I interferons (IFNs). All these complexes activate TRAF6 leading to the activation of NF-κB, IRF5 and MAPK pathways promoting the gene transcription of cell survival, proliferation, inflammation and proinflammatory cytokine production. Targeted drugs are highlighted in green. Pill icon: approved drugs and Erlenmeyer icon: preclinical studies or clinical trials.

The most relevant are: (1) the nuclear factor kappa- B cells (NF-κB) [73] and the Janus kinase/signal transducer and activator of transcription 3 (JAK-STAT3) pathways, involved in the activation, expansion and survival of cells and cytokine secretion; (2) the mitogen-activated protein kinase (MAPKs) pathway, which favors the expression of pro-inflammatory genes and (3) the interferon regulatory factor 5 (IRF5) pathway [74] that together with NF-κB pathway, promotes also the production of pro-inflammatory cytokines (Figure 1). Aside from MyD88-dependent signal transduction, TLR3 and TLR4 use the TRIF adaptor protein. TLR3 binds directly to TRIF, whereas TLR4 needs the adaptor TRAM (TRIF-related adaptor molecule). TLR3 and 4 bind to TRAF3 and TRAF6 leading to the activation of type I interferon genes and NF-κB pathway, respectively [75]. Abnormal TLR activation impairs the immune homeostasis and contributes to the onset of several inflammatory and autoimmune diseases and also some tumor malignancies [76,77]. In CLL, the BCR and TLRs from B cells recognize autoantigens and bacterial components [73]. This activation could explain, at least partially, why CLL patients have severe infections and autoimmune complications [78].

### 2.1. Recurrent Mutations and Alterations in TLR Pathway

Gain of function mutations in TLR pathway produce an increase on cell proliferation, cell survival and cytokine production in absence of cognate ligands, which results in a better supportive tumor microenvironment [79,80]. Plasma from CLL patients contains high levels of unmethylated DNA, which can trigger antilogous TLR9 activation, with the promotion of CLL cell activation and trafficking to lymphoid tissues [81].

In CLL, *MYD88* is mutated in 2% to 5% of cases [1,16]. The most frequent mutation is *L265P*, the typical mutation described in other lymphoid malignancies [82], but about 15% of the mutated CLL cases harbor other *MYD88* somatic mutations (*V147L, S243N*, and *S219C*) [16]. Additionally, sporadic mutations in *IRAK1* and in TLRs (*TLR2, TLR6*) are found [16]. *MYD88* mutations are enriched among M-CLL cases. Patients harboring *MYD88* mutations are predominantly male and young, they do not present concomitant high risk mutations (e.g., *TP53, NOTCH1, SF3B1, ATM* or *BIRC3)* or adverse cytogenetics (del17p, del11q) [16]. CLL cases with *MYD88* mutations are enriched in NF-κB and STAT3 gene expression signatures and have high basal cytokine secretion [83]. Furthermore, *MYD88* mutations induce a de novo chromatin activation enriched in genes from the NF-κB pathway [20]. *MYD88* mutations are predominantly clonal and the clinical impact of this mutation is controversial [16,80,84,85].

### 2.2. Targeting TLR Pathway

Two major strategies have been described for TLRs inhibition: blocking the binding site of TLR ligands to its receptor interfering in the intracellular signaling pathway, which can be achieved by small molecule inhibitors, monoclonal antibodies, oligonucleotides, lipid-A analogs, microRNAs, and nano-inhibitors (Figure 1) [86]; or by inhibiting IRAK proteins from the Myddosome with small molecules. There is an ongoing clinical trial targeting the TLR pathway, using IRAK4 inhibitor (CA-4948) alone or in combination with ibrutinib in relapsed/refractory (R/R) hematologic malignancies (NCT03328078) [87].

ND2158 is a small molecular IRAK4 inhibitor, which exhibited robust activity in diffuse large B-cell lymphoma activated B-cell subtype presenting *MYD88* mutations [79]. In CLL cells, ND2158 decreases cell viability independently of *MYD88* mutational status and inhibits tumor proliferation promoted by the TLR agonists, resulting in blockade of NF-κB and STAT3 signaling. Furthermore, ND2158 decreases the release of inflammatory factors from monocytes, reducing their tumor protective activity. The effect of the compound has been tested in the Eµ-TCL1 adoptive transfer mouse model that mimics human CLL disease and the tumor microenvironment [88,89]. In this context, ND2158 induces a slowed down leukemia progression, a decrease in monocytes number, cytokine secretion and cytotoxic T-cell (CD8+) activity and expansion. The decrease of CD8+ effector T cells is accompanied of a decreased expression of proliferation and activation markers (CD25, CD28 and CD137) and increased expression of exhaustion markers (PD-1, TIGIT and LAG3). This exhaustion phenotype may reduce their antitumor activity [59].

TLR9 antagonists have also been used to inhibit TLR signaling (Figure 1), showing a completely blockage when these inhibitors are used in combination with ibrutinib [57]. In this way, ibrutinib inhibits BCR signaling efficaciously but partially the TLR signaling [67] being TLR9 signaling activation a common escape mechanism after treatment of CLL cells with ibrutinib and venetoclax [68].

In vitro studies have confirmed that the combination of ibrutinib with IRAK4 inhibitors show a superior antitumor activity that each compound alone [83,90]. A clinical trial combining ibrutinib and IRAK inhibitors is ongoing (NCT03328078).

## 3. MAPK Signaling

Mitogen-activated protein kinases (MAPKs) are a group of proteins that participate in the regulation of proliferation, differentiation, migration and survival of cells. The canonical activation of this pathway starts when a ligand (e.g., a cytokine, hormone or growth/differentiation factor) binds to the extracellular portion of a receptor tyrosine kinase (RTK), with the consequent phosphorylation and the activation of retrovirus-associated DNA sequences (RAS) proteins. Then, RAS activates a member of the serine-threonine kinase RAF family (BRAF), that facilitates the phosphorylation of the mitogen-activated ERK kinase (MEK) and MEK activates extracellular signal-regulated kinase (ERK), the most important kinase in the cascade that activates different transcription factors [91]. The MAPK pathway is also activated upon BCR ligation, being the magnitude of MAPK signaling activation a direct readout of BCR signaling [92]. BCR is also able to regulate phospholipase C gamma, which causes calcium mobilization; and the PI3K activation [93] (Figure 1).

### 3.1. Recurrent Mutations and Alterations in MAPK Pathway

The MAPK pathway is altered in around half of neoplasms with different frequencies across tumor types [94]. In CLL, mutations in this pathway are detected in 5–8% of cases depending on disease stage [2,5]. These mutated cases present an increased expression of MAPK pathway genes and high levels of phosphorylated ERK, a known surrogate marker of MAPK pathway activation [95]. The most frequent mutated gene is *BRAF*, detected in 2% of CLL cases [5,95,96]. Most of these mutations cluster around the kinase domain [97], but they are different to the typical *V600E* mutation seen in other malignancies [98]. In addition, other mutations in this pathway have been reported: upstream BRAF (*KITLG*, *KIT*, *PTPN11*, *GNB1*, *NRAS* and *KRAS*) and downstream BRAF (*MAP2K1* alias Mek1 and *MAP2K2* alias Mek2) [95].

Mutations in MAPK pathway are related to adverse biological features such as high expression of CD38, CD49d and ZAP-70, U-CLL [95] and trisomy 12 [99]. Patients carrying mutations in this pathway have a 5-year TTFT similar to patients with mutations associated with worse outcome (*TP53*, *ATM* or *BIRC3*), while patients presenting concomitant mutations of TP53, ATM or BIRC3 and in genes of the MAPK pathway have the worst TTFT [95]. Subclonal *BRAF* mutations also have a prognostic impact on TTFT [15] and are associated with an adverse overall survival (OS) [100]. In the CLL4 trial, mutations in *BRAF, KRAS* and *NRAS *presented a reduced OS in both FC and rituximab and FC (FCR) treatments [60]. *BRAF* mutations are associated with refractoriness to fludarabine [101] and *KRAS* mutations with resistance to chlorambucil-based chemotherapy or anti CD20-chemoimmunotherapy [62].

Regarding the new targeted therapies (Table 1), mutations in the MAPK pathway are mediators of primary resistance to PI3K inhibitors [61]. Furthermore, activation of ERK1/2 has been associated with ibrutinib resistance [102], raising the possibility that patients with mutations in this pathway might have reduced sensitivity to ibrutinib. Although overexpression of BRAF in lymphoma cells shows resistance to venetoclax, more venetoclax resistant CLL samples are needed to confirm these results [103]. In this way, lower minimal residual disease rates were seen in patients treated with venetoclax plus rituximab [54].

In addition, recurrent mutations in *EGR2* have been described in about 8% of advanced-stage patients with CLL and are related to a poor outcome [97,104]. After BCR stimulation, EGR2 is activated via ERK phosphorylation [105]. Consequently, *EGR2* mutations participate in the dysregulation of BCR signaling [97].

### 3.2. Targeting MAPK Pathway

V600 BRAF inhibitors (vemurafenib and dabrafenib) and MEK inhibitors (trametinib, selumetinib, cobimetinib and binimetinib) are used in the clinics for patients with mutations in MAPK pathway [106]. In CLL, sorafenib, a multikinase inhibitor, induces cell death independently of BRAF status, whereas incubation of *BRAF* mutated CLL cells with PLX4720, the vemurafenib progenitor [96], vemurafenib or dabrafenib [95] fails to induce cytotoxicity probably because these BRAF inhibitors are specific for V600 mutation, and this specific mutation is rare in CLL. In contrast, ulixertinib, an ERK inhibitor [107], decreases ERK phosphorylation in MAPK-mutated CLL cases [95]. Furthermore, it has been reported that the MEK1/2 inhibitor binimetinib is effective alone or in combination with venetoclax [108] and that CLL cells carrying trisomy 12 are sensitive to MEK and ERK inhibitors [109].

## 4. Notch Signaling

**Figure 2:**
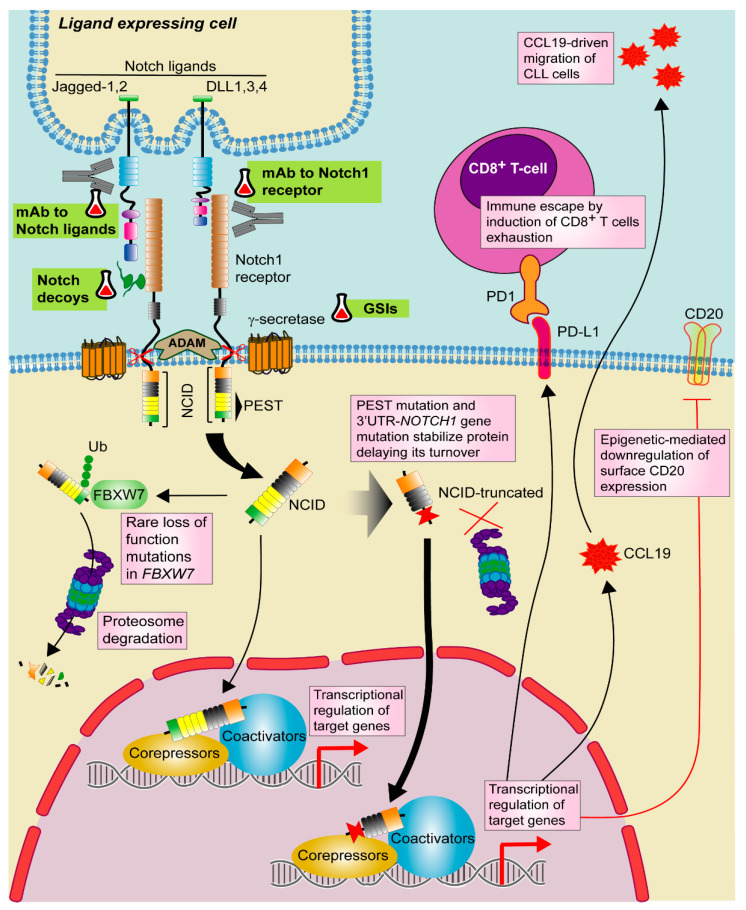
Targeting Notch1 pathway in CLL. Notch1 signaling is activated by one of the Notch ligands (DLL1,3,4 or Jagged-1,2) that binds to the Notch1 receptor on a contacting cell, with subsequent induction of a series of proteolytic events mediated by ADAM-metalloproteases and γ-secretases. These events promote the release of active Notch intracellular domain (NICD). NICD translocates to the nucleus where activates the transcription of target genes together with several transcriptional regulators. Notch1 signaling is shut down by phosphorylation of NICD and subsequent poly-ubiquitination (Ub) by F-box containing protein (FBXW7) that acts as a signal for proteasomal degradation. In CLL, recurrent mutations in *NOTCH1* PEST domain and 3′UTR stabilize the protein and delay its turnover. Although rare, loss of function mutations in *FBXW7* has been also described. In CLL, Notch1 signaling modulates CCL19-driven migration and immune escape by PD-1/PD-L1 axis as well as T-cell exhaustion. Notch1 also induces CD20 downregulation through an epigenetic mechanism. Targeted drugs are highlighted in green. Pill icon: approved drugs and Erlenmeyer icon: preclinical studies or clinical trials. GSIs: γ-secretase inhibitors, mAb: monoclonal antibody.

Notch pathway is a well evolutionary conserved signaling cascade involved in cell-fate decisions during development and the maintenance of self-renewal. Notch ligands expressed on the surface of a signal-sending cell interact with Notch receptors, which are expressed on the surface of a signal-receiving cell. There are four mammalian Notch receptors (Notch1–4) and two groups of ligands, Jagged (Jagged 1 and Jagged 2) and Delta-like (DLL1, DLL3, and DLL4) [110]. Ligand-receptor binding induces sequential cleavages by metalloproteases and γ-secretase, leading to the nuclear translocation of the intracellular Notch domain (NICD), where displaces co-repressors, recruits co-activators and finally activates the expression of several genes related to cell differentiation, proliferation and survival (Figure 2). Among the genes under direct Notch transcriptional control, note the transcriptional repressors *HES1* and *HEY1,* and the oncogene *MYC*, which contributes to the Notch-mediated transformation process of some tumors [111,112]. Physiologically, it is well-known that Notch signaling regulates T-cell commitment of common lymphoid progenitors at the expenses of B-cell differentiation. However, Notch also plays a role in B-cell development, both in the early B-cell development as well as in the differentiation toward marginal zone B cells [113].

### 4.1. NOTCH Mutations in CLL

*NOTCH1* activating mutations are one of the most recurrent alterations in CLL, accounting for 10–15% of patients at diagnosis [1,114]. *NOTCH1*-mutated patients present an adverse prognosis, high risk of transformation and poor outcome [1,115,116]. Thus, *NOTCH1* mutations prevalence increases to 20% in chemorefractory patients and up to 30% in CLL with Richter transformation (RT) [115,116,117].

Most of the mutations affect the PEST domain and result in a more stable truncated protein, with a delayed turnover that favors Notch1 signaling activation [1]. An additional hotspot in the non-coding 3′UTR of *NOTCH1* has been identified in ~3% of CLL patients, which induces the loss of the PEST domain by aberrant splicing [2]. Although rare, loss of function mutations in *FBXW7*, a ubiquitin ligase implicated in Notch1 turnover, are also found (Figure 2) [3,4,118]. Furthermore, activation of Notch1 has been described independently of the presence of *NOTCH* mutations [119].

Autocrine and paracrine mechanisms of Notch activation have been described in CLL [120]. However, *NOTCH1* mutations need the crosstalk between tumor CLL cells and surrounding cells expressing Notch ligands to trigger and sustain Notch signaling. In particular, lymph nodes would represent a specific niche for Notch activation in CLL because stromal and histiocytic cells express Notch ligands, such as DLL4 [121]. This link between Notch1 signaling and microenvironment may trigger a more aggressive behavior characterized by an increase on cell proliferation, chemotaxis and angiogenesis. In this context, Notch1 signaling regulates CLL cells migration through CCL19 and the overexpression of a genetic MYC-signature [122,123]. Accordingly, DLL stimulation triggers the expression of protumor target genes in *NOTCH1*-mutated CLL cells, together with an increase in cell proliferation, migration and angiogenesis [121].

Furthermore, Notch1 signaling promotes an immune escape mechanism in mutated CLL cells through the transcriptional regulation of HLA class-II genes and PD-L1. In particular, Notch1 up-regulates PD-L1 and impairs T-cell activation (Figure 2) [124]. A recent work showed that constitutive activation of Akt is common in *NOTCH1*-mutated CLL cells, suggesting a possible biomarker for RT [125]. Akt initiates CLL transformation via induction of Notch1 signaling between CLL cells and microenvironmental DLL1-expressing T cells.

*NOTCH1* mutations have been related to reduced benefits from anti-CD20-based chemoimmunotherapy strategies, both with rituximab [43] and with ofatumumab [24,43]. Although the effects of *NOTCH1* mutation in the pathogenesis of CLL seem mostly related to the transcriptional regulation of protumor target genes, the indirect alteration of the epigenetic environment has also been described. Thus, the resistance of *NOTCH1*-mutated CLL patients to the anti-CD20 rituximab could be likely due to the downregulation of surface CD20 expression by a HDAC-mediated epigenetic mechanism (Figure 2) [126].

Regarding the clinical impact of *NOTCH1* mutations in the response to the new targeted agents, the treatment with ibrutinib as a single agent has showed that *NOTCH1* mutation is strongly associated with lower redistribution lymphocytosis and impaired nodal shrinkage, leading to partial responses, subsequent relapses, shorter progression free survival (PFS) and OS [64]. Importantly, patients who develop RT under ibrutinib treatment have frequent adverse genomic alterations such as *TP53* and *NOTCH1* mutations [63]. In the case of venetoclax, *NOTCH1* mutation is correlated with shorter duration of response, but not probability of response [51]. Although a trend for adverse PFS and lower undetectable minimal residual disease is found in *NOTCH1*-mutated CLL cases [24,54], further validation is necessarily required in other cohorts before drawing conclusions about the effect of *NOTCH1* mutation in venetoclax-based regimens (Table 1).

### 4.2. Targeting Notch in CLL

Several strategies have been proposed for blocking Notch in hematological malignancies: targeting extracellular NOTCH1, the γ-secretase complex, Notch trafficking or Notch degradation [127]. Among them, γ-secretase inhibitors (GSIs) (Figure 2) are the most extensively evaluated drugs in different malignancies. It is known that in T-cell acute lymphoblastic leukemia (T-ALL), where more than 50% of patients have activating *NOTCH1* mutations, GSIs efficiently inhibit Notch1 oncogenic protein [128]. In CLL, they have been tested in vitro as a single agent or combined with chemotherapy [120,129]. GSI treatment abrogates drug-induced apoptosis resistance reported in *NOTCH1*-mutated CLL cells [120,129]. In addition, GSI PF-03084014 combined with fludarabine downregulates angiogenesis and CXCL12-mediated migration and invasion in *NOTCH1*-mutated CLL cells [129].

Despite promising preclinical results, the non-selectivity and the undesired gastrointestinal toxicity of GSIs promoted the development of direct strategies to block Notch1 [130], such as antibodies against the specific Notch receptors (Figure 2) [131,132,133]. Brontictuzumab (OMP-52M51) is a monoclonal antibody against human Notch1 with promising antitumor efficacy in T-ALL xenograft models [134]. In CLL and MCL cells with *NOTCH1* mutations, OMP-52M51 efficiently inhibits Notch stimulation and cell proliferation induced by DLL ligands [121,135]. Although a phase I study showed efficacy of brontictuzumab in solid tumors [136], the clinical development of this antibody has been stopped. Novel strategies such as antibodies targeting Notch ligands have also been investigated (Figure 2) [133]. In particular, dual targeting of DLL4 and VEGF induces antitumor responses in solid malignancies [137] and could be of potential interest in *NOTCH*-mutated B lymphoid malignancies. At preclinical level, other approaches have shown antitumor efficacy through Notch inhibition, such as the use of Notch decoys for either DLL-mediated or Jagged-mediated signaling as well as the use of natural products with ability to modulate Notch signaling (Figure 2) [133,138]. In CLL, another strategy that has been recently proposed for the therapeutic use of Notch targeted agents is its combination with ibrutinib. At biological level, Notch1 and BCR pathways are functionally linked, being *NOTCH1*-mutated CLL cells more responsive to BCR signaling [139]. This strategy could be particularly relevant in patients with RT, a subgroup with limited therapeutic options [117].

## 5. RNA Splicing

**Figure 3:**
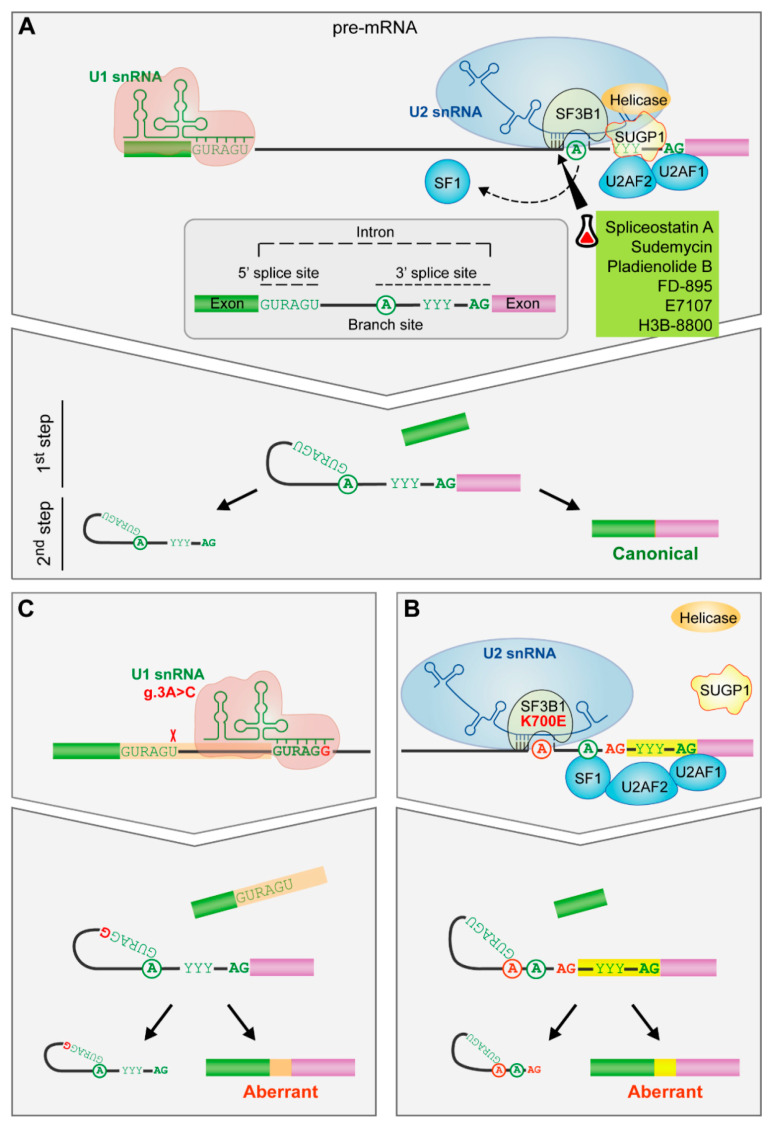
The impact of spliceosome mutations in the spliceosome assembly. (**A**) Spliceosome assembly of U1 and U2 snRNPs. The spliceosome removes the internal sequences (introns) of the pre-mRNA and splices together the remaining fragments (exons) after the recognition of conserved sequences found at the 5′ and 3′ end of the intron and the branch site (an adenosine residue). U1 snRNP recognizes the 5′splice site by base pairing interactions. Then, SF1, U2AF2 and U2AF1 recognize the branch site, the polypyrimidine tract and the AG dinucleotide of the 3′ splice site, respectively. Afterwards, SF3B1 helps in the recognition of the branch site by U2 snRNA. Finally, the subsequent recruitment of other snRNPs will drive to the splicing of the intron. (**B**) *SF3B1* mutations. *SF3B1* mutations disrupt interactions with SUGP1 spliceosomal protein and contribute to an upstream alternative 3′ splice site usage through the use of an alternative branch site. (**C**) *U1* snRNA mutation. The A > C change in the third position favors the C-G base pairing between the third position of the U1 snRNA and the 5′splice site leading to the formation of aberrant 5′ splice sites. Targeted drugs are highlighted in green. Erlenmeyer icon: preclinical studies or clinical trials. R: purine, Y: pyrimidine.

The spliceosome removes non-coding fragments (introns) from messenger RNA precursors (pre-mRNA) through two catalytic steps necessary for the expression of most eukaryotic genes (Figure 3A). Alternative splicing is key for the regulated generation of different mRNA transcripts and protein variants from a single gene. The spliceosome is a dynamic and complex molecular machinery that consists of more than 150 polypeptides and five small nuclear ribonucleoproteins (snRNP: U1, U2, U4, U5 and U6) each of which is composed of one small nuclear RNA (snRNA) and some associated proteins [140].

U1 snRNP takes part in the first step of spliceosome assembly, recognizing the 5′ splice site through base pairing interactions involving the 5′ end of U1 snRNA and sequences at the 5′ end of the intron. Then, U2 snRNP binds to the branch site -an intronic adenosine involved in the first chemical step of the splicing reaction- through base pairing interactions between U2 snRNA and nucleotides flanking the branch site adenosine. This helix, characterized by the bulge out of the branch site adenosine, is recognized by SF3B1 protein, a key component of U2 snRNP, involving a conformational change that ultimately facilitates the approaching of the branch site adenosine to the 5′ splice site and the first catalytic step of the splicing reaction [141,142] (Figure 3A).

### 5.1. Recurrent Splicing Factor Mutations

Mutations in the spliceosome components have a role in cancer [143]. In CLL, mutations in *SF3B1* and *U1* have been described [3,144]. *SF3B1* mutation disrupts interactions with SUGP1 spliceosomal protein, which is involved in the branch site recognition complex, contributing to the activation of an upstream alternative 3′ splice site through the use of an alternative branch site [145,146,147,148] (Figure 3B).

Approximately 50% of the aberrant RNA transcripts undergo nonsense mediated decay, resulting in downregulation of canonical transcripts [145,149]. In addition, full-length transcript analyses have shown downregulation of retained introns (i.e., enhanced splicing) in *SF3B1* mutated patients [150].

*SF3B1* is mutated in 8–21% of CLL patients [2,5]. Most of the mutations are missense and they are localized in the highly conserved C-terminal domain of SF3B1, composed of multiple HEAT repeats, being *K700E* the most frequently mutated site (50% of reported cases) [151]. *SF3B1* mutations are associated with advanced stages, male sex, high leukocyte counts, elevated B2 microglobulin levels, high CD38 expression, U-CLL subgroup, intermediate CLL epigenetic subgroup, stereotyped BCR subset 2 and R110-mutated IGLV3-21 subgroup, as well as with 11q deletion and fludarabine resistance [11,13,152,153,154,155].

*SF3B1*-mutated CLL patients present CD20 downregulation and, similarly to *NOTCH1*-mutated cells, they also present high levels of active intracytoplasmic Notch1 accompanied by a *NOTCH1*-related gene set enrichment and overexpression of a DVL2 isoform, involved in the Wnt pathway and Notch1 signaling repression [156,157]. Additionally, other gene set enrichments including “Cytokine-cytokine receptor interaction” and “Phosphatidylinositol signaling system” have been associated with *SF3B1* mutations [158]. *SF3B1*-mutated patients have a shorter PFS [159] and OS [3,153]. If the subclonal architecture of the tumor is considered, *SF3B1* mutations with a variant allele frequency (VAF) > 12% predict for shorter TTFT [15,17]. But even patients with a low VAF show a shorter PFS after chlorambucil or chlorambucil and ofatumumab treatment [24] (Table 1). One-third of CLL patients resistant to BTK inhibitors therapy develop RT with a dismal outcome [160] and in this group, *SF3B1* mutations are more frequent than *BTK* mutations [24], the most common ones described in ibrutinib resistant patients [161].

Recently, an A > C mutation in the third position of U1 snRNA has been described, which changes the preferential A–U base-pairing between U1 snRNA and the 5′ splice site to C–G base-pairing and thus, creates novel splice junctions, some of them in known cancer drivers (Figure 3C). *U1* mutation is present in 3.8% of CLL patients, is associated to U-CLL subtype and confers an adverse prognosis with a shorter TTFT [144]. Notably, none of the samples with *U1* mutation shows *SF3B1* mutations [144]. Although they appear at very low frequency, mutations in additional components of U2 snRNP (*U2AF2*, *SRSF1*, *SRSF7*, *RBMX*, and *ZRSR2*) and other splicing factors have been described in CLL, as well as mutations in genes involved in RNA transport and metabolism [162].

### 5.2. Targeting the Spliceosome in CLL

Small-molecule splicing modulators bind to SF3B1, preventing the recognition of the branch site [163,164] (Figure 3A). These bacterial fermentation products and synthetic derivatives show antitumor properties. FR901464 derivatives, such as spliceostatin A and sudemycin, cause more cytotoxicity in primary CLL samples than in healthy B lymphocytes inducing apoptosis by the switch of *MCL1* splicing toward its proapoptotic isoform [165,166]. In an adoptive transfer mouse CLL model, sudemycin D6 decreases the number of CLL primary cells in the peripheral blood and in the spleen [165]. Importantly, the combination of sudemycin D1 with ibrutinib shows an enhanced in vitro cytotoxicity [165] and so do the combinations of spliceostatin A and ABT-199 (venetoclax) or spliceostatin A and ABT-263 [166].

Pladienolide B and its derivative FD-895 also induce more apoptosis in CLL cells than in healthy lymphocytes by inducing a pattern of intron retention [167]. E7107, derived from pladienolide B, reprograms apoptosis, decreases Mcl-1 dependence and increases Bcl-2 dependence in CLL. This compound sensitizes primary CLL cells to venetoclax treatment and reverts venetoclax resistance in CLL-like cells from Eµ-TCL1–based adoptive transfer murine model [168]. E7107 entered two phase I clinical trials in advanced solid tumors achieving stable disease (NCT00499499) and partial response (NCT00459823) as the best tumor response. These studies have been discontinued because two patients presented vision loss and another one bilateral optical neuritis [169,170]. H3B-8800, an orally available pladienolide B derivative, has preferential cytotoxic effect on spliceosome-mutant epithelial and myeloid malignancies [171] and also on CLL cells (manuscript in preparation). A phase I clinical trial (NCT02841540) evaluates H3B-8800 in patients with myelodysplastic syndromes, acute myeloid leukemia or chronic myelomonocytic leukemia. Preliminary results show dose-dependent target engagement and predictable pharmacokinetic profile and safety even with prolonged dosing. Though no complete or partial responses have been achieved, decreased red blood cell or platelet transfusion requirements have been observed in 14% of enrolled patients [172].

## 6. Conclusions

CLL is a heterogeneous disease both at molecular and clinical level. Current genomic studies have identified novel mutated genes affecting important biological pathways, including TLR, MAPK and Notch signaling as well as RNA splicing. Some of these alterations contribute to the development and progression of CLL through specific mechanisms of action that include their relationship with the tumor microenvironment. In recent years, data on the prognostic value of these mutations have been gradually emerging. Given the low frequency of these mutations, only through the efforts of the entire scientific community we will unravel the real prognostic and predictive impact of these mutations. The development of more specific targeted therapies and novel combination treatments will help to design personalized effective treatments and strategies to improve the outcome of patients with CLL.

## Data Availability

Not applicable.
